# Developmental outcomes in a child with corpus callosum abnormalities and congenital heart disease after Cuevas Medek Exercises: A case report

**DOI:** 10.1002/ccr3.4637

**Published:** 2021-08-16

**Authors:** Gabriela Ramires de Oliveira, Marcelo Fabris Vidal

**Affiliations:** ^1^ Physiotherapy Department at Fisioterapia Neurofuncional Clinic Porto Alegre Brazil; ^2^ Physiotherapy Department at Anatomy Rehab Dubai UAE

**Keywords:** case report, congenital heart disease, corpus callosum abnormalities, motor development, rehabilitation

## Abstract

Cuevas Medek Exercises (CME) stimulate active exercises, minimize handlings, and harness scientific parameters to promote neuroplasticity, shifting paradigms in pediatric rehabilitation. Distinct CME protocols had positive outcomes in a child with corpus callosum abnormalities and comorbidities indicating its potential as a research topic and in treating developmental motor delays.

## Graphical abstract

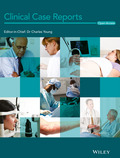

## 1 INTRODUCTION

Corpus callosum abnormalities (CCA) are rare congenital anomalies characterized by the complete or partial absence of the corpus callosum.^1^, ^2^ Patients with CCA may present with an isolated anomaly or an anomaly associated with other central nervous system (CNS) malformations and/or somatic anomalies.^1^, ^2^ The variable presence of other associated anomalies could explain the heterogeneous clinical symptoms and neurological outcomes in children with CCA, ranging from no symptom to mild or severe neurodevelopmental disabilities.^2^

A recent meta‐analysis of prenatal studies revealed that approximately two‐thirds of children with isolated agenesis of the corpus callosum showed a normal neurodevelopmental outcome.^2^ However, among those with CCA and comorbidities, 60% presented with developmental motor delay.^2^

Extracallosal brain abnormalities were found in 46% of CCA cases and were associated with a high incidence of gross motor delay.^3^ Colpocephaly, which is an abnormal enlargement of the occipital horn of the lateral ventricle associated with motor abnormalities, is frequently observed in patients with CCA.^3^, ^4^, ^5^ According to Puvabanditsin et al.,^5^ 91% of colpocephaly cases presented with developmental motor delay.

The involvement of other systems apart from the CNS is found in 81% of CCA cases.^3^ This is the primary determinant of poor neurodevelopmental outcomes in children prenatally diagnosed with CCA.^2^ Congenital heart disease (CHD), which occurs in 21% of CCA cases, is a common non‐CNS comorbidity.^3^ According to the American Heart Association and the American Academy of Pediatrics guideline, patients with CHD are already at high risk for developmental delay.^6^, ^7^ In a systematic review, Snookes et al.^8^ concluded that children with CHD were at increased risk for impaired neurodevelopment regardless of their lesion, and they typically presented with worse motor outcomes than healthy children. Howell et al.^7^ reported that the risk of severe motor impairment was 11 times greater in children who underwent surgical intervention within the first year of life.

During infancy, gross motor delays typically appear as the primary manifestations of altered neurodevelopment.^9^ Longitudinal studies investigating motor development in children with CHD revealed a delay in gross motor skill acquisition in children as young as 2 months old, and these delays were consistent up to 2 years of age.^9^ At older ages, 39%–42% of children with CHD presented with persistent gross motor deficits.^9^

Gross motor functioning is critical to overall physical functioning, and depending on the severity of motor impairments, it may affect psychosocial function as well.^9^, ^10^ Therefore, to prevent delay in children at risk for developmental motor delay, early physiotherapy intervention targeting the achievement of developmental motor milestones with a focus on neuroplasticity is imperative.^2^, ^9^

Practicing goal‐oriented tasks involving active movements at a high intensity is considered an integral part of effective interventions because they result in experience‐dependent neuroplasticity.^11^ These features are included in Cuevas Medek Exercises (CME), which have recently been shown to effectively prevent developmental motor delay because it harnesses optimal evidence‐based parameters to promote neuroplasticity.^12^ CME therapy is a physiotherapy approach to treat children with developmental motor delay by impacting CNS.^13^ According to Ramon Cuevas, who developed the therapy, CME are mainly based on the principle of provoking novel automatic motor reactions using exercises against gravity with progressive distal holding.^13^ The therapy is used in almost all parts of the world, including well‐known rehabilitation centers in the United States, Canada, and Australia, and it has attracted growing interest from researchers, clinicians, and patients. However, there is scarce scientific evidence to justify it as a treatment option.

Therefore, our primary goal was to measure the effect of a CME‐based intervention on a child presenting with developmental motor delay due to CCA, colpocephaly, and CHD. Our secondary aim was to evaluate the effectiveness of the CME‐based intervention as an intensive protocol and as a standard protocol in the same case.

## 2 CASE PRESENTATION

A 9‐month‐old girl presented to the physiotherapy department due to developmental motor delay. At 37 gestational weeks, prenatal ultrasonography showed dilated brain ventricles. She was delivered at 40 gestational weeks through C‐section after 16 hours of labor and fetal distress. At 9 days of age, she underwent brain magnetic resonance imaging (MRI), which showed dilation of bilateral lateral ventricles, most prominently at the atrium, temporal horn, and occipital horn, as well as the thinning of the corpus callosum. At 8 months of age, the neurologist referred her to physiotherapy due to concerns related to developmental motor delay caused by CCA and colpocephaly. At 9 months of age, she underwent a physiotherapy assessment based on anamnesis, physical examination, and a complimentary assessment using the CME motor scale^13^ and the Alberta Infant Motor Scale (AIMS).^14^

The CME motor scale is composed of 41 items for assessing motor development through automatic motor reactions. The response to each item is assessed using a 4‐point scale; a score of 0, 1, 2, and 3 indicates no response, an initiated reaction, an incomplete reaction, and a complete reaction, respectively. The sum of the scores of all items is divided by a constant value to reach the maximum CME motor scale age of 16 months. Assessed motor skills and CME motor scale age are used to create a personalized treatment plan and to measure the effectiveness of intervention.^13^

The AIMS is a gross motor observational tool for evaluating the activity of antigravitational muscles in 58 postures. One point is scored for each posture observed. A maximum score of 58 points indicates complete motor development. The AIMS is a validated scale used in clinical practice and research. It can detect developmental delays or abnormalities, identify mild changes in motor development, and measure the effectiveness of intervention.^14^

The assessments were conducted before and after each treatment phase by two experienced physiotherapists. One of the physiotherapists was responsible for the treatment. The other one was not involved in the treatment and assessed the child using pre‐recorded videos. The scores assigned by both the physiotherapists were compared, and the lowest score was considered in case of a discrepancy.

### 2.1 Treatment

The intervention was based on the concepts of CME.^12^, ^13^ Exercises aimed at achieving missed developmental milestones, and they were practiced actively. Although exercises targeting developmental milestones at the expected ages were started in order to build a biomechanical foundation, more complex functions were also addressed to improve basic motor skills.^12^, ^13^ Therefore, when trunk control was expected, standing and walking exercises were started. When standing was expected, walking exercises were practiced. Our primary aim was to promote motor development of an advanced level in order to achieve developmental milestones quickly. Each exercise was repeated up to eight times; the difficulty level of each exercise was increased in each session. Handling was gradually reduced while moving the hands from the proximal to distal part of the child's body, which increased independent reactions. Static stretching is not included in the concept of CME although stretching is embedded within functional movement.^12^, ^13^

The intervention was divided into three phases: intensive protocol, home‐program protocol, and standard protocol.

During the intensive protocol, the child underwent four weeks of physiotherapy intervention; 45‐minute sessions were conducted twice daily five days per week for the first two weeks. The frequency was reduced to once per day during the third week in order to lessen the intensity of exercise and to avoid fatigue. Then, in the fourth and final week, the frequency was again increased to twice per day. Exercises focusing on sitting, transferring from prone and supine to sitting, crawling, transferring from sitting and crawling to standing, standing, and walking were started. Her parents were taught the exercises to ensure daily stimuli at home. The intensive protocol was conducted between the ages of 9 and 10 months, and she was reassessed at the end of this period.

After the first phase of treatment, she was diagnosed with CHD. According to the cardiac ultrasonography report, she presented with moderate to severe mitral stenosis due to supravalvar mitral membrane, mild left atrial enlargement, and elevated right ventricular and pulmonary artery pressure. At 14 months of age, she underwent cardiac surgery to remove supravalvar mitral membrane. There were no complications during the surgery and recovery period.

Seven months elapsed between the diagnosis of CHD and complete recovery allowing her to continue physiotherapy intervention at the clinic. Therefore, a home‐program protocol was initiated immediately following the intensive protocol. Exercises targeting creeping, crawling, transferring from kneeling to standing, and standing self‐supported on a horizontal surface were taught to the parents. Exercises and progressions were frequently reported using videos to the physiotherapist. This protocol was conducted between 10 and 17 months of age.

She resumed her physiotherapy two months after the cardiac surgery. After reassessment, she underwent a standard protocol consisting of 45‐minute sessions twice per week. Exercises to stimulate transferring from crawling to standing, standing, and walking were performed during this time. Daily exercises of the home‐program protocol were emphasized. This protocol was conducted between 17 and 20 months of age, and she was reassessed at the end of the protocol.

### 2.2 Outcomes

**FIGURE 1:**
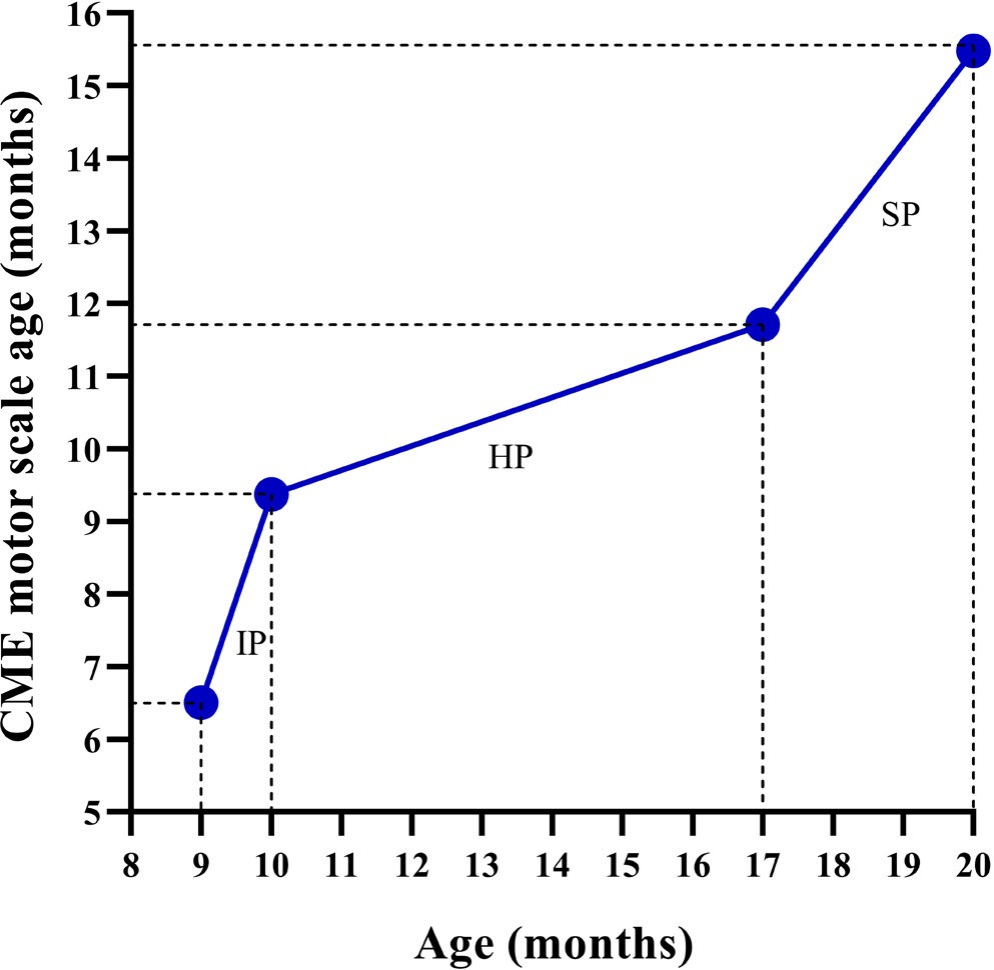
Comparison between the child's CME motor scale age and chronological age. CME: Cuevas Medek Exercises, IP: intensive protocol, HP: home‐program protocol, and SP: standard protocol

**FIGURE 2:**
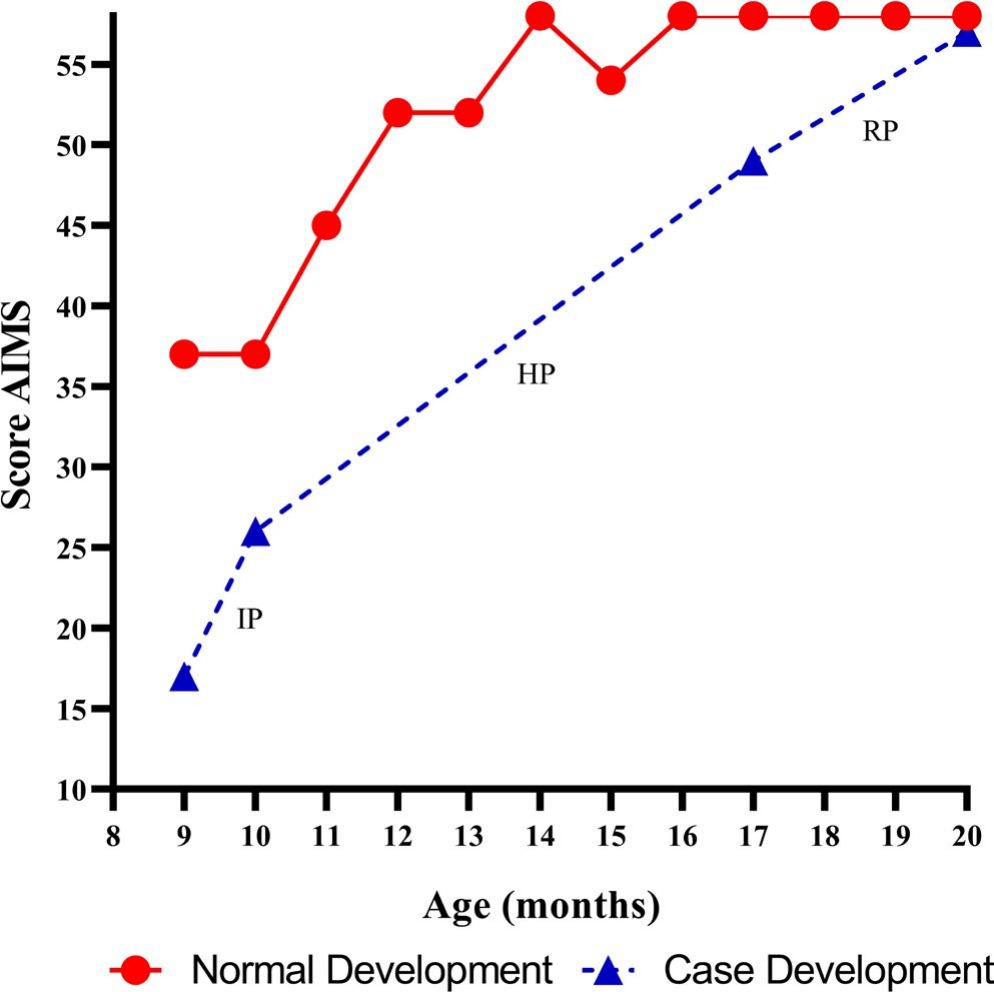
Comparison between the normal motor development curve according to the AIMS^14^ and the child's AIMS scores at different ages. Scores below the red line indicate abnormal motor development. Scores above the red line indicate normal motor development. AIMS: Alberta Infant Motor Scale, IP: intensive protocol, HP: home‐program protocol, and SP: standard protocol

At baseline, a mild spasticity in the right side of her body was the only observed abnormality. Her CME motor scale age was 6.5 months (Figure 1), and she scored 17 points on the AIMS (Figure 2).

After one month of the intensive protocol consisting of 35 sessions, at the age of 10 months, she reached a CME motor scale age of 9.37 months (Figure 1) and scored 26 points on the AIMS (Figure 2). The child improved head control, began to roll over, started to sit unsupported, began to transfer from supine to sitting independently, improved standing with support at one forearm and self‐supported standing on horizontal surfaces, started to stand self‐supported against vertical surfaces, and began to take steps with support at one forearm.

After seven months of the home‐program protocol, at 17 months of age, her CME motor scale age was 11.71 months (Figure 1), and she scored 49 points on the AIMS (Figure 2). Her functional improvements included crawling, transferring from kneeling to standing with support on a horizontal surface, and beginning to walk self‐supported on horizontal surfaces.

Following three months of the standard protocol consisting of 28 sessions, at the age of 20 months, she achieved a CME motor scale age of 15.48 months (Figure 1) and scored 57 points on the AIMS (Figure 2). Her functional achievements included transferring from crawling to standing, unsupported standing, and walking. She also began to step over obstacles and walk up and down 10‐centimeter steps.

## 3 DISCUSSION

A CME‐based intervention was effective for achieving motor milestones and preventing developmental motor delay in a child presenting with CCA, colpocephaly, and CHD although the child was at high risk for anomalous motor development due to her health conditions.

After one month of the intensive protocol, the child showed an increase of 2.87 months in her CME motor scale age (Figure 1). She achieved a CME motor scale age of 9.37 months, which was slightly less than her chronological age of 10 months. However, despite an increase of 9 points on the AIMS, she scored 11 points less than the value associated with the normal motor development at her age; this placed her development on an abnormal motor performance curve (Figure 2). On average, children with CCA and comorbidities begin to roll over at 8.9 months of age, sit unsupported at 13.1 months of age, and stand while holding on to something at 18.7 months of age.^15^ In our case, the child was doing these functional activities at 10 months of age. At the end of the intensive protocol, her development was within the normal range for the achievement of developmental motor milestones by healthy children proposed by the World Health Organization (WHO).^16^

Following the home‐program protocol, she achieved a CME motor scale age of 11.71 months at the chronological age of 17 months (Figure 1). Her development was approximately 4 months delayed compared to the expected 16 months for the complete motor development on the CME motor scale. On the AIMS, she showed an improvement of 23 points. However, she scored 9 points less than the value associated with the normal motor development at her age; this placed her development on an abnormal motor performance curve (Figure 2). On average, children with CCA and comorbidities begin crawling at 19.4 months of age,^15^ whereas the child reported in this study began crawling at 16 months of age. Additionally, according to the WHO,^16^ she achieved all developmental motor milestones expected for healthy children of her age.

After three months of the standard protocol, her CME motor scale age increased by 3.77 months. She achieved a CME motor scale age of 15.48 months, which was just under the expected 16 months for the complete motor development on the CME motor scale (Figure 1). On the AIMS, she scored 1 point less than the expected score of 58 points for complete motor development (Figure 2). On average, children with CCA and comorbidities begin to stand up and walk independently at 24 and 28.3 months of age, respectively.^15^ In our case, the child who received treatment based on the concepts of CME started to stand independently at 17 months of age and walk independently at 18 months of age. These results also placed her development within the normal range for the achievement of developmental motor milestones by healthy children proposed by the WHO.^16^

The use of a CME‐based intervention may explain these functional improvements. Following an early CME‐based intervention, a normal motor development was achieved by a child with hydrocephalus who was at high risk for anomalous development.^12^ According to a study by Ramires de Oliveira and Fabris Vidal,^12^ CME use optimal scientific parameters, including intensity, repetition, progression, meaningful exercises, and enriched environment, to promote neuroplasticity. Moreover, CME are based on the concepts of stimulating active movements using gradually minimized handling during meaningful and progressively more difficult exercises.^12^, ^13^ A recent systematic review claimed that high‐intensity task‐ and goal‐oriented exercises using self‐generated movements were common characteristics of the most effective interventions for improving motor development.^11^

Comparisons between the improvements in her motor development assessed after each protocol showed that regular intervention conducted by a physiotherapist was more effective than a home‐program protocol, even when the physiotherapist monitored the progression closely using video. However, the child's improvements during the home‐program protocol highlighted the importance of family involvement and engagement in the intervention in order to provide the stimuli required for motor development at home and improve treatment outcomes.

A comparison between the results of the intensive protocol and the standard protocol revealed that both the protocols were effective for improving motor development. The rate of improvement per month assessed based on the scores on the CME motor scale and AIMS, indicates that the intensive protocol was slightly more effective than the standard protocol. However, it is important to note that the number of treatment sessions in one month of the intensive protocol was larger than that in three months of the standard protocol. Cope and Mohn‐Johnsen^17^ reported moderate evidence indicating that a longer therapy time may be slightly more beneficial for improving motor function in children with cerebral palsy than a shorter therapy time. Nevertheless, there is insufficient evidence to determine whether a high‐frequency therapy is more effective than a low‐frequency therapy.^17^ Cope and Mohn‐Johnsen^17^ justified the use of intensive therapies not to induce a large treatment effect, but to accelerate the progress toward treatment goals.

## 4 CONCLUSION

A CME‐based intervention was effective for achieving motor milestones and preventing developmental delay in a child at high risk for anomalous development due to CCA, colpocephaly, and CHD. Furthermore, an intensive protocol showed fast improvements in this case. However, additional studies are required in order to further validate the effectiveness of a CME‐based treatment and to develop an ideal treatment protocol. Nevertheless, a CME‐based intervention may be considered as a topic for further study and as an intensive or standard therapy option to decrease or prevent developmental motor delay.

## CONFLICT OF INTEREST

The authors declare that there is no conflict of interest.

## AUTHOR CONTRIBUTIONS

Gabriela Ramires de Oliveira contributed to treatment, data collection, data analysis, and manuscript writing. Marcelo Fabris Vidal contributed to treatment, data collection, data analysis, and manuscript writing.

## ETHICAL STATEMENT

This case report was written based on patient's treatment data, preserving child's anonymity. Standard patient consent form was explained and signed by the child's responsible prior to manuscript submission.

## Data Availability

Data available on request due to privacy/ethical restrictions.
